# Biochemical and Molecular Analysis of Field Resistance to Spirodiclofen in *Panonychus citri* (McGregor)

**DOI:** 10.3390/insects13111011

**Published:** 2022-11-02

**Authors:** Lu-Yan Cheng, Dong-Yuan Hou, Qin-Zhe Sun, Shi-Jiang Yu, Si-Chen Li, Hao-Qiang Liu, Lin Cong, Chun Ran

**Affiliations:** 1 Citrus Research Institute, Southwest University/Chinese Academy of Agricultural Sciences, National Engineering Research Center for Citrus, Chongqing 400712, China; 2 Key Laboratory of Entomology and Pest Control Engineering, College of Plant Protection, Southwest University, Chongqing 400716, China

**Keywords:** detoxification, spirodiclofen, *Panonychus citri*, cytochrome P450s

## Abstract

**Simple Summary:**

The citrus red mite, *Panonychus citri*, is one of the most destructive citrus pests worldwide. Spirodiclofen is an important chemical acaricide to control *P. citri*. In this study, the complete *acetyl-CoA carboxylase* (*ACCase*) gene between field spirodiclofen-resistant and laboratory-susceptible strains of *P. citri* were sequenced and compared based on the mode of action of spirodiclofen and the reported literature. Enzyme activity measurement and digital gene expression profile analysis were applied to identify candidate metabolic resistance genes. RT-qPCR and RNA interference (RNAi) further confirmed that the most prominent upregulated gene, *CYP385C10*, may be involved in the field of spirodiclofen-resistance of *P. citri* in China. These results provide the molecular foundation for understanding the spirodiclofen resistance in *P. citri*.

**Abstract:**

Spirodiclofen is one of the most widely used acaricides in China. The citrus red mite, *Panonychus citri* (McGregor) (Acari: Tetranychidae), is one of the most destructive citrus pests worldwide and has developed a high resistance to spirodiclofen. However, the molecular mechanism of spirodiclofen resistance in *P. citri* is still unknown. In this study, we identified a field spirodiclofen-resistant strain (DL-SC) that showed 712-fold resistance to spirodiclofen by egg bioassay compared to the susceptible strain. Target-site resistance was not detected as non-synonymous mutations were not found by amplification and sequencing of the *ACCase* gene of resistant and susceptible strains; in addition, the mRNA expression levels of *ACCase* were similar in both resistant and susceptible strains. The activity of detoxifying enzymes P450s and CCEs in the resistant strain was significantly higher than in the susceptible strain. The transcriptome expression data showed 19 xenobiotic metabolisms genes that were upregulated. Stage-specific expression profiling revealed that the most prominent upregulated gene, *CYP385C10,* in transcriptome data was significantly higher in resistant strains in all stages. Furthermore, functional analysis by RNAi indicated that the mortality caused by spirodiclofen was significantly increased by silencing the P450 gene *CYP385C10.* The current results suggest that overexpression of the P450 gene, *CYP385C10*, may be involved in spirodiclofen resistance in *P. citri*.

## 1. Introduction

Phytophagous mites of the *Tetranychus* and *Panonychus* are severe pests for plants worldwide [1,2]. Among these, the citrus red mite, *Panonychus citri* (McGregor) (Acari: Tetranychidae), is one of the most notorious and devastating citrus pests, causing a huge fruit yield loss every year [3,4,5,6]. Acaricides are crucial in managing *P. citri* [7], which is unlikely to be changed shortly. However, frequent and long-term use of acaricides has caused *P. citri* to rapidly develop resistance [8], posing a severe challenge to effectively controlling *P. citri* in agriculture. To maintain mite populations below economic thresholds, it is crucial to design and apply effective resistance management measures to retain the efficacy of the existing acaricide portfolio [9].

The acaricide spirodiclofen, which belongs to the family of spirocyclic tetronic acids [10], interferes with lipid biosynthesis through potent inhibition of acetyl-coenzyme A carboxylase (ACCase) [11,12,13]. It can make it efficient against spider mites in all stages of development, including eggs, with minimal acute toxicity to adults but a significant influence on female fertility [14,15]. Spirodiclofen was first introduced to the Chinese market in 2005 and is still valuable for managing *P. citri* in China [16,17]. According to the Institute for the Control of Agrochemicals, Ministry of Agriculture, P. R. China (ICAMA), 203 commercial products of spirodiclofen have been registered against *P. citri* [18]. However, frequent use of spirodiclofen or increasing the dose/concentration speeds up the development of the spirodiclofen-resistance in phytophagous mites [19]. Field populations of phytophagous mites were resistant to spirodiclofen in some areas in the USA, Germany, Brazil, China, Turkey, and Iran [20,21,22,23,24,25,26,27,28]. 

Understanding the molecular mechanism of acaricide resistance is the basis for developing resistance management strategies [29]. The molecular mechanisms resulting in cyclic ketoenol acaricides and insecticides resistance in some insect and mite pests have been extensively studied, including target-site and metabolic resistance. Karatolos et al. (2012) found that a single substitution of glutamic acid to lysine (E645K, *Trialeurodes vaporariorum* numbering) in *ACCase* was associated with decreased spiromesifen efficacy in the greenhouse whitefly *T. vaporariorum* [30]. The A1079T variant in ACCase (tetur21g02170) was observed between the laboratory spirodiclofen resistance strain (SR-VP) and the laboratory spirodiclofen sensitivity strain Lon-Inb in *T. urticae* [31,32]. ACCase protein was more abundant in a laboratory-selected spirotetramat resistance *Aphis gossypii* strain [33]. The same authors’ further study found a combination of *ACCase* overexpression and 14 non-synonymous mutations were associated with low to moderate levels of spirotetramat resistance of *A. gossypii* [34,35]. The A2083V variant in *ACCase* was observed between high levels of ketoenol resistance in *Bemisia tabaci* strains and the susceptible strain [36]. These studies suggested the possible existence of target sites of the *ACCase* gene in insect and mite pests for ketoenol resistance.

Relative to target resistance, metabolic resistance caused by enhanced detoxification is another important mechanism of insecticide resistance. According to synergist experiments and enzyme assays, the resistance of mite pests to spirodiclofen could be associated with P450s, CCEs, and Glutathione-S-transferases (GSTs) [28,37,38,39,40]. Overexpressed P450 and CCE genes are associated with spirodiclofen resistance in *T. urticae* and *Panonychus ulmi* [41,42,43]. Wybouw et al. (2019) used quantitative trait locus (QTL) to map spirodiclofen resistance in *T. urticae* pointed toward 3 QTLs, including clustered and copy variable cytochrome P450 genes and NADPH cytochrome P450 reductase [31]. Heterologous expression of *CYP392E10* of *T. urticae* can metabolize spirodiclofen [41]. Pan et al. (2018) speculated that overexpressed *CYP380C6* is possibly involved in spirotetramat resistance in *A. gossypii* [44]. These studies suggested the detoxification enzymes P450s, CCEs, and GSTs might play crucial roles in the ketoenol resistance of insect and mite pests.

Digital gene expression profiling analysis has been successfully applied to screen the function of genes and is a robust next-generation high-throughput sequencing technology for understanding gene expression and structure [45]. In agricultural pest control, digital gene expression profiling is crucial for elucidating resistance mechanisms [46,47]. It has been widely used to examine gene expression patterns in mites [42,48,49,50]. In 2021, the whole genome of *P. citri* was sequenced and made to be available to the public [51]. Genome-based digital gene expression profiling allows us to better study spirodiclofen-resistant mechanisms in *P. citri*.

Here, we established a field spirodiclofen-resistant strain to elucidate the resistance mechanism in *P. citri*. The complete *ACCase* gene between spirodiclofen-resistant and laboratory-susceptible strains of *P. citri* was sequenced and compared. The activity difference of P450s, CCEs, and GSTs between resistant and sensitive strains was analyzed. Genome-based digital gene expression profiling data were applied to uncover candidate metabolic resistance genes. Additionally, we showed that a P450 gene, *CYP385C10*, is the most prominently upregulated in both transcriptome data and RT-qPCR. RNAi data suggested that the overexpression of *CYP385C10* may be involved in the field of spirodiclofen-resistance of *P. citri* in China. These results provide the molecular foundation for spirodiclofen resistance of *P. citri*.

## 2. Materials and Methods

### 2.1. Spider Mites and Chemicals

The laboratory susceptible-strain (LS) was initially collected from a wild-collected *P. citri* population from the Citrus Research Institute (Chongqing, China) [52], and DNA from this strain was used for *P. citri* genome sequencing [51]. The DL-SC is a field strain, initially collected in 2019 from a citrus orchard in Danling Sichuan Province, China, and highly resistant to spirodiclofen with a spirodiclofen resistance ratio of 162 compared to the LS strain. The DL-SC strain was further maintained with 5 g L^−1^ spirodiclofen on all life stages on citrange plants sprayed with a hand-pressurized sprayer until runoff after transfer to our laboratory. Mites of the LS and DL-SC have been reared on potted citrange plants in a climatically controlled incubator at 25 ± 1 °C, 70–80% relative humidity (RH), and 16: 8 h light:dark photoperiod. 

Commercial formulations of Spirodiclofen (Envidor, 240 g L^−1^ SC medium; Bayer Crop Science (Hangzhou, China)) were used in toxicity bioassays.

### 2.2. Egg Bioassays

Egg bioassays were performed in a standard format as previously described [39]. Briefly, 30 adult females were transferred to a 2-cm-edge square smooth citrus leaf disc on wet cotton wool and permitted to lay eggs for 6–8 h. After the removal of the adults, the eggs were sprayed with 1 mL spray fluid at 1.45 × 10^5^ pa pressure. To determine the final spirodiclofen concentrations, pre-experiments were carried out to obtain a series of solution concentrations in an appropriate range. The plates were then placed in a climatically controlled room at 26 ± 0.5 °C, 60% RH, and 16:8 h light:dark photoperiod. Three replicates of five concentrations plus a distilled water control treatment were used for the bioassays. Mortality was assessed after 11 days when adults appeared in control. Control mortality never exceeded 10%. 

### 2.3. Sequencing of ACCase Gene from Susceptible and Resistant Strains

Total RNA was isolated from the following two samples: LS and DL-SC. Approximately 300 adult females were homogenized for each sample by liquid nitrogen grinding. According to the manufacturer’s recommendations, total RNA was extracted from the sample using the ReliaPrep^TM^ RNA Tissue Miniprep System (Promega). *ACCase* gene was identified in the *P. citri* proteome of the LS strain using a BLASTp approach (*P. citri* gene IDs encoding these subunits can be accessed at https://ngdc.cncb.ac.cn/search/?dbId=gwh&q=GWHBAOM00000000&page=1 (accessed on 6 June 2021)). Blastp further confirmed the *ACCase* gene in the National Center for Biotechnology Information (NCBI) database (http://www.ncbi.nlm.nih.gov (accessed on1 July 2021)). Based on the sequence, a set of primers was designed to amplify the complete *ACCase* gene [53] open reading frame in overlapping fragments (Table S1). A quantity of 1 μg total RNA for each sample was reverse transcribed into cDNA using HiScript III 1st Strand cDNA Synthesis Kit (+gDNA wiper) (Vazyme, Nanjing, Jiangsu, China). In total, 1 μL of complementary DNA (cDNA) was used as a template for PCR carried out in a Biometra Tone 96 PCR (Analytik Jena). PCR reactions were performed in 50 mL containing 25 μL of 2 × Phanta Max Master Mix (Vazyme), 10 μM of each primer, under the following conditions: 3 min at 95 °C, 35 cycles of 15 s at 95 °C, 15 s at 55 °C, the 90 s at 72 °C and a final extension of 5 min at 72 °C. Tsingke Biological Technology sequenced the PCR products. To search for possible mutations in the *P. citri ACCase*, the assembled nucleotide and deduced amino acid sequences of spirodiclofen-resistant and susceptible strains were aligned with those of a broad range of organisms using BioXM2.7 software. *ACCase* nucleotide sequences of *P. citri* strains LS and DL-SC were submitted to the GenBank database (GenBank accession numbers MZ855509, and MZ855510, respectively).

### 2.4. Measurement of Detoxifying Enzyme Activities

Carboxylcholinesterases (CCEs). The CCEs activity assay kit (Beijing Solarbio Science & Technology Co., Ltd., Beijing, China) was used to determine the total esterase enzyme activity. Using 1-Naphthyl acetate as substrate, the detailed procedure was performed according to the manufacturer’s instructions. After the reaction stopped, the OD value was immediately measured at 450 nm by a microplate reader. The results were determined based on the standard curve of 1-Naphthyl acetate and protein concentration.

Glutathione S-transferases (GSTs). The GSTs activity assay kit (Beijing Solarbio Science & Technology Co., Ltd., Beijing, China) was used to determine the total Glutathione S-transferases enzyme activity. Using CDNB and GSH as substrates, the detailed procedure was performed according to the manufacturer’s instructions. After the reaction stopped, the OD value was immediately measured at 340 nm by a microplate reader. The results were determined based on the protein concentration of the enzyme source, and specific activity was converted from OD value.

Cytochrome P450 monooxygenases (P450s). Shang’s method was used to test P450s activity [54]. Using 4-nitroanisole as the substrate to determine the O-demethylase ability, the detailed procedure was performed in a standard format as previously described by Wang et al. [55]. After the reaction stopped, the OD value was immediately measured at 400 nm by a microplate reader. The results were determined based on the standard curve of nitrophenol and protein concentration. 

Deutonymphs of the LS and DL-SC were placed in three 1.5 mL enzyme-free tubes, respectively. The number of mites in each tube was approximately 500 and homogenized by liquid nitrogen grinding. The crude enzyme was extracted from the six samples using PBS (0.1 mol L^−1^, pH 7.8) and collected using single enzyme-free tubes. The total protein concentrations were tested with the Bradford method using bovine serum albumin [56]. Three biological and three technical replicates were analyzed for the experiment.

### 2.5. Sample Collection, RNA Extraction, and Transcriptome Sequencing

Adult female mites of LS and DL-SC were placed on water-treated citrus leaf discs to deposit eggs for 8 h, after which they were removed. Developing mites were collected in the deutonymphal stage for RNA-seq analysis. For each of the LS and DL-SC strains, about 500 deutonymphs were collected as a sample (one biological replicate). The collected mites were frozen in liquid nitrogen and stored at −80 °C for RNA extraction. Three biological replicates represented each strain. Total RNA was extracted as described above.

After the total RNA had been extracted, the quantity and quality were assessed with the Agilent 2100 Bioanalyzer (Agilent Technologies) and RNase-free agarose gel electrophoresis. The enriched mRNA was then fragmented into short fragments using fragmentation buffer and reverse transcribed into cDNA using random primers. The first-strand cDNA was synthesized with random hexamer primers, and the complementary strand cDNA was synthesized using the mixture of DNA polymerase I, RNase H, dNTP, and buffer solution. The double-stranded cDNA fragments were purified using the QiaQuick PCR extraction kit (Qiagen), end-repaired with an added poly(A), and ligated to Illumina sequencing adapters, which were performed by Guangzhou Genedenovo Biotechnology Co., Ltd. (Guangzhou, China). The ligation mixture was size selected by agarose gel electrophoresis, PCR amplified, and sequenced on the Illumina HiSeq™ 2500 (Illumina, San Diego, CA, United States) by paired-end reads (150 bp) mode and performed at the Gene Denovo Biotechnology Co. (Guangzhou, China). The raw reads were filtered to remove adaptors, low-quality sequences, and ploy-N to obtain high-quality clean reads. After Q10, Q20, and Q30, GC content and sequence duplication level of the high-quality clean data were simultaneously calculated, and the high-quality clean reads were mapped to the reference genome (https://ngdc.cncb.ac.cn/search/?dbId=gwh&q=GWHBAOM00000000&page=1 (accessed on 2 August 2021)) with TopHat v2.0.12 [57] in default parameters. Besides, we used HTSeq v0.6.1 to count the number of reads mapped to each gene and calculated fragments per kilobase of transcript sequence per million base pairs sequenced (FPKM) for each gene based on the length of the gene and the read counts mapped to the gene [58]. Cufflinks v2.1.1 was used to identify novel transcripts.

DEseq software was used to identify the Differential gene expression between resistant and susceptible strains. EdgeR software was used to determine the Fold-change (FC) values and false discovery rates (FDR). Genes with a cut-off of FDR ≤ 0.05 and absolute log2 (FC) ≥ 1 in DL-SC relative to LS were significantly differentially expressed. The Gene Ontology (GO) enrichment analysis of differentially expressed genes (DEGs) was implemented by the GO seq R package, in which the gene length bias was adjusted. The Kyoto Encyclopedia of Genes and Genomes (KEGG) database was used to determine Pathway assignments. The KEGG Orthology-Based Annotation System (KOBAS) software tested for statistically significant DEGs enrichment in KEGG pathways. Significantly enriched GO terms and KEGG pathways of the genes in the biological functions of a module relative to the background were defined using the hypergeometric test with a false discovery rate threshold of <0.05 [59]. The raw data were submitted to the National Center for Biotechnology Information (NCBI) Sequence Read Archive (SRA) database with the accession number SRP333472.

### 2.6. Real-Time Quantitative PCR (RT-qPCR)

Adult female *P. citri* mites of the LS and DL-SC were placed on citrus leaf discs to deposit eggs to assess gene expression levels. Approximately 2000 eggs, 1000 nymphs, and 100 adults were collected for each sample to quantify the expression of genes in LS and DL-SC strains. Each sample had three biological replicates. Total RNA was extracted, and 1 µg of total RNA was used to synthesize the first-strand cDNA described above. Two stable reference genes, *ELF1A* (HM582444) and *GADPH* (HM582445) were used to normalize the results [60]. Primers for the genes of interest and a set of two reference genes are listed in Table S2. The RT-qPCR reactions were performed on a qTOWER³G qPCR system (Analytik Jena) using the ChamQ™ Universal SYBR^®^ qPCR Master Mix (Vazyme) according to the manufacturer’s instructions. A 10-fold dilution series of pooled cDNA was used to assess the efficiency of the RT-qPCR reaction for each gene-specific primer pair. A no-template control (NTC) was also included to detect possible contamination. Experiments were performed using three biological and three technical replicates for each gene. A dissociation curve analysis was performed to check for the presence of a single amplicon. 

### 2.7. Double-Stranded RNA Synthesis and RNA Interference

The P450 gene *CYP385C10* (sequence fragment: 472 bp) and the enhanced green fluorescent protein (EGFP) gene (NCBI: JQ064510, negative control, sequence fragment: 540 bp) were amplified by PCR using primers containing the T7 RNA polymerase promoter. Primers of the dsRNA are presented in Table S2. The Transcript Aid T7 High Yield Transcription Kit (Thermo Scientific, Waltham, MA) was used to synthesize dsRNA according to the manufacturer’s instructions. The dsRNAs were further purified following Li et al. [61]. Additionally, dsRNA concentration was determined with the Agilent 2100 Bioanalyzer (Agilent Technologies), and dsRNA integrity was verified by 1% agarose gel electrophoresis. 

A leaf-disc dsRNA feeding method was used to knock down the expression of targeted genes, as previously described by Li et al. [61]. In brief, citrus leaves were cut into 4-cm^2^ sections, dehydrated at 60 °C for 4 min, and then treated for 6 h with dsRNA (1000 ng μL^−1^). After dsRNAs were fully absorbed, the leaves were placed on wet filter paper. The 1-day-old protonymph mites of DL-SC (starved for 5 h) were separately placed on each leaf disc (100 per disc) and incubated at 26 ± 0.5 °C with 60 RH and a 16:8 h light:dark photoperiod. After 24 h of feeding, RNAi effectiveness was evaluated by RT-qPCR to determine the reduction in transcription levels. 

After 24 h of feeding, the surviving protonymph mites of DL-SC on additional replicate leaf discs were transferred onto leaf discs treated with a 4800 mg L^−1^ dose of spirodiclofen with the same conditions described earlier. The dsEGFP and water were controls. The number of living and dead mites was counted after adults appeared in control. Three biological replicates and three technical replicates were conducted.

### 2.8. Statistical Analysis

Lethal concentrations (LC_50_, slopes, and 95% confidence limits) were calculated with Abbott’s formula [62]. Resistance ratios (RRs) were calculated by dividing the LC_50_ value of the resistant strain by that of the susceptible strain.

Statistical calculations were descriptive statistics and were carried out using SPSS 20.0 software. Gene expression levels were quantified using the comparative threshold cycle (2−^ΔΔCt^) method [63]. The relative quantities of expression levels of genes and detoxifying enzyme activities in the LS and DL-SC strains were determined by an independent-sample *t*-test at the 0.05 level. RNAi knockdown efficiencies and mortality rates were evaluated using one-way analyses of variance, followed by Duncan’s multiple tests, with a significance level of *p* < 0.05.

## 3. Results

### 3.1. Spirodiclofen Efficacy against the Susceptible and Resistant Strains

The lethal concentration 50 (LC_50_) value of the LS egg, derived from log-dose probit–mortality data, was 20.09 mg L^−1^ (Table 1). The LC_50_ value of the field-collected DL-SC resistant strain was 14,300 mg L^−1^, and the resistance ratio (RR) was 712. In addition, the response of LS and DL-SC to spirodiclofen appeared to be very heterogeneous (the slope of the probit line was 1.53 and 1.27, respectively).

### 3.2. Amplification, Sequencing, and Expression Analysis of ACCase in Susceptible and Resistant Strains of P. citri

To investigate spirodiclofen resistance mediated by target site insensitivity, full-length complementary DNA (cDNA) fragments of the *ACCase* gene encoding a putative spirodiclofen target were cloned from the LS and DL-SC strains, and their amino acid sequences were compared. Aligning the *ACCase* (2318 amino acids) of DL-SC (MZ855510) against the *ACCase* of LS (MZ855509) revealed that they had almost the same amino acid sequence as the *ACCase* reported from *P. citri* (GenBank accession number KJ675439). No amino acid sequence polymorphisms were found in *ACCase* between the LS and DL-SC strains.

**Figure 1:**
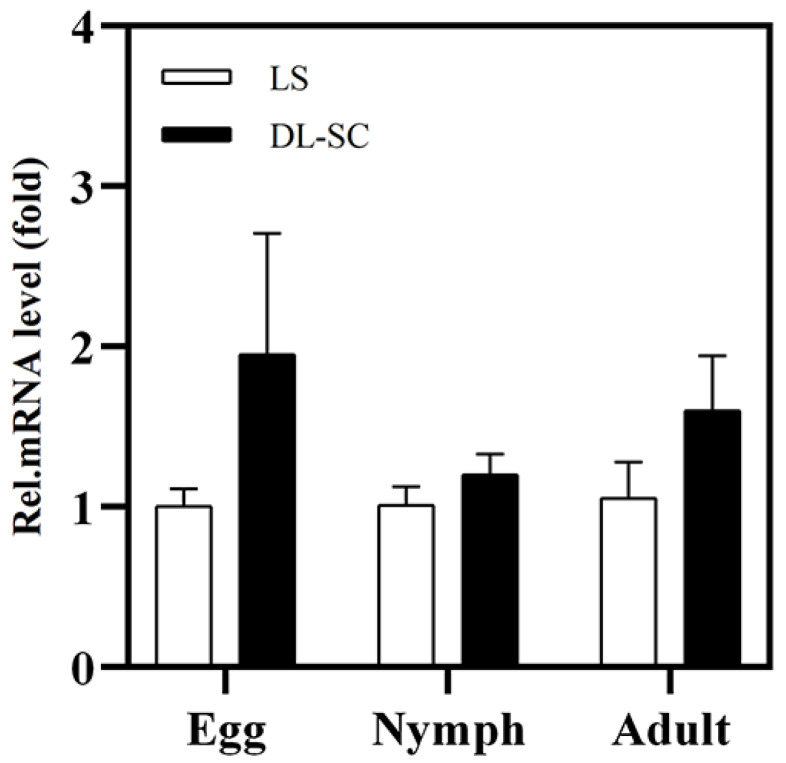
RT-qPCR expression analysis of *ACCase* gene expression in different life stages between the LS and DL-SC strains. Error bars represent the standard deviation of the calculated mean based on three biological replicates. In each panel, means without asterisks indicate not significantly different (*t*-test, *p* > 0.05).

To determine the possibility of upregulation of *ACCase* in the DL-SC strain, the mRNA levels of age-synchronized life stages were determined by RT-qPCR. However, a slight upregulation in the *ACCase* mRNA levels of age-synchronized eggs, nymphs, and adults was observed in the DL-SC strain, with no significant difference (Figure 1).

### 3.3. Activity of Detoxifying Enzymes in LS and DL-SC

To determine the relationships between the detoxifying enzyme activity and spirodiclofen resistance, enzymatic activity assays were performed. Table 2 shows the specific activities of P450s, CCEs, and GSTs in LS and DL-SC. The activity of P450s in DL-SC (1.47 ± 0.10 nmol/mg protein/min) was significantly higher than in LS (0.96 ± 0.10 nmol/mg protein/min). The change in the activity of CCEs was similar to that of P450s, i.e., compared with LS (31.26 ± 1.02 nmol/mg protein/min), and the activity was significantly higher in DL-SC (47.66 ± 1.38 nmol/mg protein/min). The specific activity of GSTs in the DL-SC strain (1.49 ± 0.38 μmol/mg protein/min) was higher when compared with the LS strain (1.40 ± 0.03 μmol/mg protein/min) but not significantly different.

### 3.4. Transcriptome Sequencing

The six independent cDNA libraries (LS and DL-SC) constructed from the two *P. citri* strains were sequenced on the Illumina sequencing platform. They generated a mean of 6.6 Gb of clean data for each library (Table 3). The Q20 and Q30 values were >97% and >93%, respectively, and the GC content ranged from 36 to 37%, indicating that the data quality was sufficient for transcriptome analysis.

### 3.5. Identification and Analysis of the Differentially Expressed Genes

The gene expression level was compared between the susceptible strain LS and the resistant strain DL-SC based on FPKM values. The false discovery rates (FDR) < 0.05 and |log_2_ fold change (FC)| ≥ 1 were set as the threshold for significantly differential expression. In total, 1618 genes were identified as significantly differentially expressed genes (DEGs) between susceptible and resistant strains (Figure S1). Among DEGs, the number of upregulated genes (825 genes) was greater than the number of downregulated genes (793 genes). The log_2_ (FC) was from −17.66 to 17.76.

GO enrichment analysis provides all GO terms significantly enriched in DEGs compared to the genome background and filters the DEGs corresponding to biological functions. After GO enrichment analysis, DEGs were enriched into 53 functional groups in the following 3 main categories: ‘biological process’ (657), ‘cellular component’ (591), and ‘molecular function’ (619). The subcategories ‘cellular process’, ‘metabolic process,’ and ‘single-organism process’ contained most of the genes within the biological process class. In the ‘cellular component’ class, the subcategories ‘cell,’ ‘cell part,’ and ‘organelle’ held enormous numbers of genes. ‘Binding’ and ‘catalytic activity’ were the most numerous subcategories in the ‘molecular function’ category (Figure S2). 

**Figure 2:**
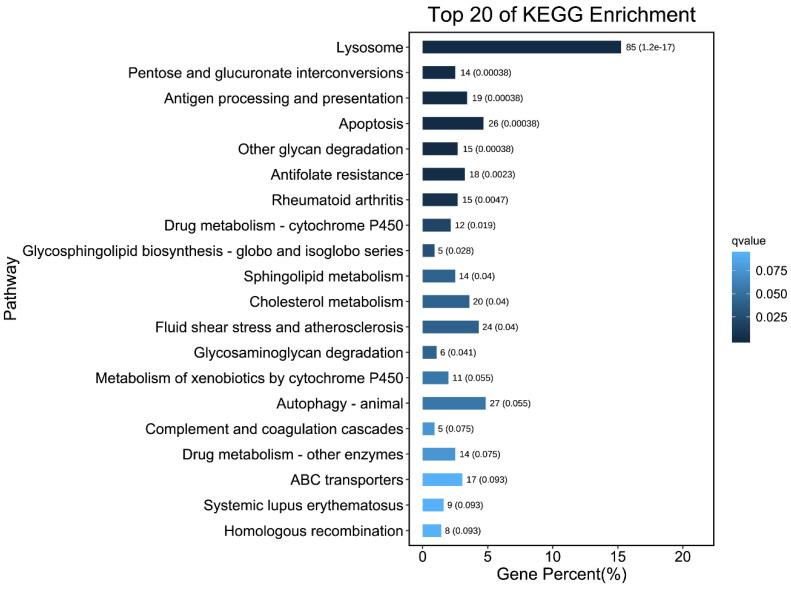
Top 20 KEGG classification of differentially expressed genes. The abscissa values mean the number of genes annotated to the pathway and their proportion to the total number of genes annotated, and the ordinate represents the different KEGG classifications. The q value is defined by the ratio of the differentially expressed genes enriched in the pathway and the number of all genes enriched in the same pathway (*p* < 0.05).

The 557 DEGs are annotated and classified in the KEGG database, distributed over 320 pathways. The most abundant pathway was ‘lysosome’ (85 genes, 26.56%), followed by ‘Autophagy’ (27 genes, 8.44%). In addition, the KEGG pathway enrichment analysis showed that DEGs were significantly enriched in 20 pathways (Figure 2). The pathway ‘glycosphingolipid biosynthesis globo and isoglobo series’ had the highest enrichment factor value, containing 5 genes (*EVM0000504*; *EVM0003878*; *EVM0008164*; *EVM0010891*; *EVM0011065*). In addition, KEGG analysis revealed that the pathways of ABC transporters, drug metabolism-cytochrome P450, and xenobiotic metabolism via cytochrome P450 were among the top 20 enriched pathways for the DL-SC compared to the LS, with 17, 12, and 11 significant DEGs, respectively. These genes may participate in the process of detoxification of acaricides in mites.

Arthropods have evolved a mechanism for reducing exposure by responding to changes in the quantity or quality of major detoxifying enzymes and transporters [64]. Major detoxification enzymes include P450s, CCEs, and GSTs. Important transporters are the ATP-binding cassette (ABC) protein family, the major facilitator family (MFS), intradiol ring-cleavage dioxygenases (ID-RCDs), and UDP-glycosyl transferases (UGTs). The 825 upregulated DEGs included 19 xenobiotic metabolisms that contained genes, which included six P450 genes, seven ABC genes, three CCE genes, two MFS genes, and one UGT gene. Information on these genes is presented in Table 4.

### 3.6. Validation of the Overexpression of Metabolism-Related Genes Using RT-qPCR

**Figure 3:**
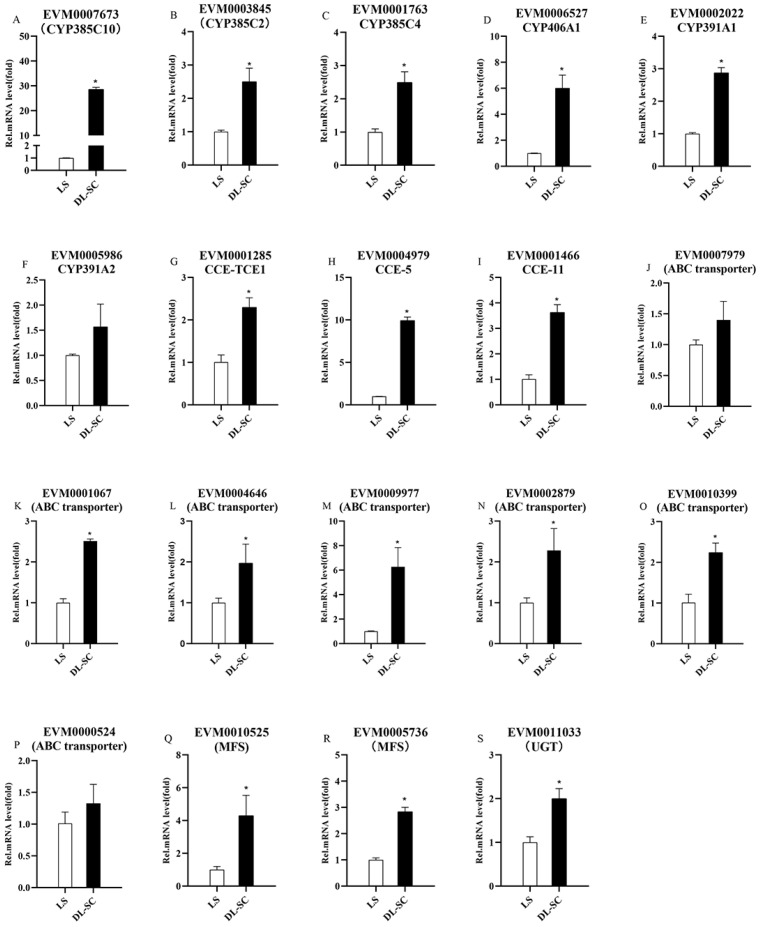
Overexpressed metabolism-related genes’ relative expression between the LS and DL-SC strains. (**A**–**F**) Relative expression of the P450 genes; (**G**–**I**) Relative expression of the CCE genes; (**J**–**P**) Relative expression of the ABC transporters; (**Q**–**R**) Relative expression of the major facilitators; (**S**) Relative expression of the UGT gene. The expression levels were normalized to that of LS. Error bars represent the standard deviation of the calculated mean based on three biological replicates. In each panel, means with asterisks indicate significantly different (*t*-test, *p* > 0.05).

To verify the transcriptome expression results, 19 upregulated metabolism-related genes’ relative expression in LS vs. DL-SC was performed on cDNA from the two strains’ deutonymphs by RT-qPCR (Figure 3). Relative to their expression levels in LS, the expression levels of 6 P450 genes, *EVM0007673* (*CYP385C10*), *EVM0003845*, *EVM0001763*, *EVM0006527*, *EVM0002022*, and *EVM0005986* were upregulated by 28.64-, 2.51-, 2.49-, 6.01-, 2.88-, and 1.57-fold in DL-SC, respectively. Relative to their expression levels in LS, 3 CCE genes *EVM0001285*, *EVM0004979*, and *EVM0001466* were upregulated by 2.30-, 9.95-, and 3.63-fold in DL-SC, respectively. Relative to their expression levels in LS, 7 ABC genes *EVM0007979*, *EVM0001067*, *EVM0004646*, *EVM0009977*, *EVM0002879*, *EVM0010399*, and *EVM0000524* were upregulated by 1.40-, 2.51-, 1.98-, 6.27-, 2.28-, 2.24-, and 1.33-fold in DL-SC, respectively. Relative to their expression levels in LS, two MFS genes and one UGT gene, *EVM0010525*, *EVM0005736*, and *EVM0011003*, were upregulated by 4.29-, 2.84-, and 2.00-fold in DL-SC, respectively. 

### 3.7. Overexpression of CYP385C10 in Different P. citri Life Stages

**Figure 4:**
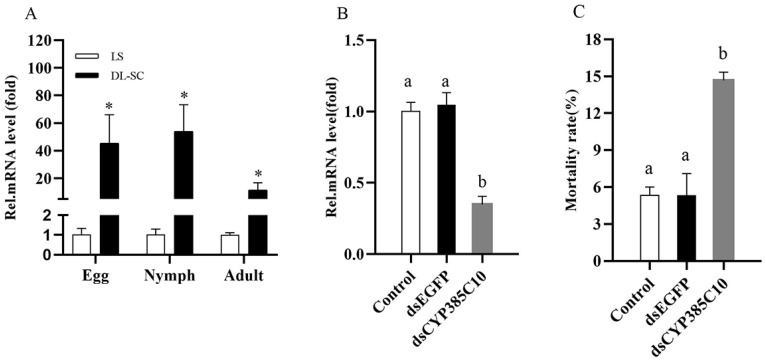
*CYP385C10* is involved in spirodiclofen resistance. Relative expression of *CYP385C10* at different life stages (**A**). RT-qPCR analysis of *CYP385C10* expression after feeding dsRNA in the spirodiclofen-resistant strain of *P. citri*, relative to expression in control (**B**). Mortality after feeding with *CYP385C10* dsRNA in the spirodiclofen-resistant strain (**C**). Error bars represent the standard error of the calculated mean based on three biological replicates. In each panel, means with asterisks indicate significantly different (*t*-test, *p* > 0.05), and means with different letters are significantly different (Duncan test, *p* < 0.05).

The enzymatic assays and the transcriptome expression results have indicated the involvement of both P450s and CCEs in spirodiclofen resistance in *P. citri*. Consequently, we further screened the most significantly overexpressed P450 gene *CYP385C10* in the resistant strain (compared to the susceptible strain) to investigate the expression in different life stages and RNAi analysis. An RT-qPCR analysis of different life stages was performed on cDNA from the two strains’ eggs, nymphs, and adults. The expression levels of *CYP385C10* were upregulated by 45.42-, 54.08-, and 11.42-fold in eggs, nymphs, and adults of DL-SC, respectively, as compared to LS (Figure 4A).

### 3.8. Functional Analysis of CYP385C10 via RNAi

After the DL-SC protonymph was fed on the double-stranded RNA (dsRNA) of *CYP385C10* for 24 h, the expression level of *CYP385C10* was significantly decreased (65.21%) compared to the control (Figure 4B), indicating an effective silencing of *CYP385C10* by RNAi in *P. citri*.

After DL-SC protonymphs had fed on the dsRNA of *CYP385C10* for 24 h, they were exposed to spirodiclofen (4800 mg/L) for 72 h, and the mortality caused by spirodiclofen was significantly increased (Figure 4C).

## 4. Discussion

Spirodiclofen is the most important acaricide applied worldwide to control phytophagous mites [38]. However, the control efficiency of this acaricide decreased rapidly due to resistance development. Field populations of *T. urticae*, *P. ulmi*, and *P. citri* have acquired resistance to spirodiclofen [20,21,24,26,27,28]. Unlike *T. urticae* and *P. ulmi*, *P. citri* has not reported molecular mechanisms of spirodiclofen resistance. To verify the mechanism of spirodiclofen resistance in this species, we selected a Chinese field population with a high dose of spirodiclofen and obtained a resistant strain named DL-SC. Although this strain was originally collected from a field with a different genetic background than the laboratory sensitivity strain LS, the strain-specific mechanisms can also contribute to the resistant study. In this study, bioassays were carried out on eggs for spirodiclofen, an ovicidal acaricide. The LC_50_ value of spirodiclofen was 14,300 mg L^−1^, and the resistance ratio was 712. The level of spirodiclofen resistance in the eggs of this study was different from observations made in *T. urticae* and *P. ulmi*; eggs from *T. urticae* and *P. ulmi* strains expressing high spirodiclofen resistance in larvae were susceptible to moderate resistance to spirodiclofen [26,39]. Different sources of resistant materials may cause the difference. The egg toxicity bioassay data showed that the strain DL-SC develops a high level of resistance against spirodiclofen.

Previous studies reported the *ACCase* gene might be possibly correlated with ketoenol resistance in *T. urticae*, *B. tabaci*, *A gossypii*, and *T. vaporariorum*. To investigate whether *ACCase* mediates target site insensitivity of spirodiclofen resistance of *P. citri*, the *ACCase* gene of *P. citri* was completely sequenced in overlapping fragments in susceptible and resistant strains. A comparison of full-length nucleotide and amino acid sequences revealed that, despite several synonymous mutations, no fixed non-synonymous mutations were present in DL-SC compared to LS. Besides, we did not detect any differences in mRNA levels of age-synchronized eggs, nymphs, or adults between susceptible and resistant strains. In *T. urticae*, although the genomic and quantitative basis of resistance to spirodiclofen was linked to the A1079T variant in *ACCase* [31], there is no further evidence of a causal role of *ACCase* in spirodiclofen resistance in *T. urticae*. Besides, QTLs are relatively large genomic regions; other genes or non-coding genetic changes may cause spirodiclofen resistance in *T. urticae* [36]. Similarly, although the TaqMan allelic discrimination assay confirmed the E645K mutation in *ACCase* played a potential role in the spiromesifen resistance of *T. vaporariorum* [30]; furthermore, the study failed to associate the frequency of the mutation in this species with spiromesifen resistance in field populations from southern Greece [65]. Thus, it is plausible that A1079T and E645K variants in *ACCase* are not bona fide ketoenol resistance mutations [36]. A recent study showed the A2083V variant in *ACCase* is the possibility of alternative/additional mutation(s) or additional mechanism(s) associated with or contributing to the ketoenol resistance phenotype [36]. Additionally, a combination of *ACCase* overexpression and non-synonymous mutations contributes to low to moderate levels of spirotetramat resistance in *A. gossypii* [66]. Although our present results showed resistance to spirodiclofen in *P. citri* of China is not likely to be caused by the change in *ACCase*, the gene is still important in insects for ketoenol resistance. Target-site resistance in the spirodiclofen resistance of *P. citri* should be further studied. 

Given that target-site-based resistance mechanisms could not be detected in the field of spirodiclofen-resistant strains (DL-SC). Therefore, we used enzymatic assays combined with RNA sequencing in DL-SC and LS strains to help explain whether metabolic mechanisms determine spirodiclofen resistance in *P. citri*. In the enzymatic assays, the total CCEs and P450s activities were detected significantly higher in DL-SC than in LS, indicating that they might be involved in spirodiclofen resistance in *P. citri*. This was similar to the enzymatic assay results obtained with spirodiclofen-resistant *T. urticae* [38,39]. Besides, we compared Illumina RNA-Seq data of the field-resistant strain (DL-SC) and the susceptible strain (LS) of *P. citri*. We identified 825 upregulated and 793 downregulated DEGs. Upregulated DEGs related to pesticide resistance and host plant adaptation were identified in DL-SC, ranging from classical detoxification enzymes and transporters such as P450s and CCEs to major facilitator transporters. Our results, however, did not find GST genes among the upregulated DEGs. Enzymatic assays combined with RNA sequencing results did not indicate the involvement of GST enzymes in the spirodiclofen resistance of *P. citri*, which differs from the results of Lv et al. [67], who found that the upregulated GST genes *TuGSTd1* and *TuGSTd2* are likely to contribute to spirodiclofen resistance in *T. urticae*. The difference in the results obtained in the study of Lv et al. [67] and the current study is consistent with spirodiclofen resistance in *T. urticae* [38] and *P. ulmi* [21], indicating that the spirodiclofen resistance mechanism is complex and may differ among spider mite strains.

For spider mites, P450s are documented to be involved in spirodiclofen resistance. Previous studies showed that the inhibitor piperonylbutoxide (PBO) could enhance the toxicity of spirodiclofen in resistant strains (*T. urticae*, *P. citri*, and *P. ulmi*), suggesting P450s were involved in spirodiclofen resistance in both tetranychid species [28,37,39]. Genome-wide gene expression analysis of two high-level spirodiclofen *T. urticae*-resistant strains revealed several P450 genes overexpressed, including *CYP392E7* and *CYP392E10*; further functional expression confirmed that *CYP392E10* could metabolize spirodiclofen [41]. Furthermore, spirodiclofen resistance was mapped in a cluster of overexpressed P450 genes using a genomic and quantitative approach employing an SR-VP-derived inbred line [31]. Bajda et al. (2015) provide a comprehensive strand-specific RNA-seq-based transcriptome resource for *P. ulmi* derived from susceptible and spirodiclofen-resistant strains (PSR-TK); many detoxification genes were identified, including P450 genes [42]. In our enzymatic assay, the activity of P450s in DL-SC was higher than in LS. We, therefore, suspect that the spirodiclofen resistance of the DL-SC strain of *P. citri* in China is mediated by the overexpression of P450 genes. Our study found six P450 genes upregulated in DL-SC after transcriptome analysis, and these genes were clustered into Clan3 and Clan4 [51]. In *T. urticae*, all the upregulated P450 genes responsible for spirodiclofen resistance were clustered into the CYP2 clan [31,41,68]. We speculate that different mite species may cause the difference. It has been reported that the levels of P450 genes usually change during the different developmental stages of most insects [69]. The most significantly overexpressed P450 gene, *CYP385C10*, was significantly higher in the resistance strain in all stages. In addition, we found that RNAi silencing of *CYP385C10* significantly increased the mortality of the DL-SC. This result is consistent with a report in *A. gossypii*; suppression of *CYP380C6* increased the sensitivity of resistant aphids to spirotetramat [44]. Therefore, we speculate that *CYP385C10* might be important for spirodiclofen resistance and suggest that the function of *CYP385C10* should be investigated with heterologous expression systems as previously undertaken for *CYP392E10*, which metabolizes spirodiclofen [41]. In summary, the present result shows that detoxification enzymes P450s were involved in an enhanced spirodiclofen metabolism in the field spirodiclofen-resistant strain of *P. citri*.

Next to P450s, studies showed that detoxification enzymes CCEs are also associated with spirodiclofen resistance in *T. urticae* and *P. ulmi* [27]. Previous synergism and enzymatic assays pointed out that CCEs involved spirodiclofen resistance in *T. urticae*, which was subsequently held the involvement of *CCE04* in spirodiclofen resistance seem more likely to quantitative differences based on kinetic enzyme data [43]. RNA-seq data from the spirodiclofen susceptible and resistant strains of *P. ulmi* revealed multiple CCEs were differentially expressed [42]. In recent research, significantly higher activity levels of esterases were found in Iranian field *P. ulmi* Urmia and Shahin Dej populations, which indicates CCEs may be involved in spirodiclofen resistance in Iranian *P. ulmi* [27]. In our study, the activity of CCEs in DL-SC was significantly higher than in LS, and three esterase genes were upregulated in DL-SC. The results suggest that the potential involvement of CCEs in spirodiclofen resistance in *P. citri* cannot be ruled out.

In addition to the P450 and CCE families, we also found some other genes of xenobiotic metabolism are upregulated. Studies showed that UGTs might play important roles in forming insecticide/acaricide resistance [70,71,72]. Several UGT genes in acaricide resistance have been functionally proven in abamectin-resistant strains of *T. urticae* [73] and *Tetranychus cinnabarinus* [74]. In our study, the UGT gene was upregulated in DL-SC. Similar to the spirotetramat resistance in *A. gossypii*, UDP-glucose 6-dehydrogenase protein was more abundant in the resistant strain [33]. According to several studies, the resistance of various insects to pesticides is associated with ABC transporters [66,75,76,77,78]. In our study, multiple ABC transporters were upregulated in DL-SC. Although studies suggest that ABC transporters in spider mites may be linked to the formation of pesticide resistance [42,79,80,81,82], direct functionally validated studies are still absent.

## 5. Conclusions

Our study investigates the molecular mechanisms of spirodiclofen resistance in *P. citri*. The target-site resistance is not detected after complete mite *ACCase* sequencing. Enzymatic assays indicated that P450s and CCEs potentially involved spirodiclofen resistance in *P. citri*. The transcriptome and RT-qPCR showed that *CYP385C10* is the most prominent upregulated gene in the spirodiclofen-resistant strain. RNAi data suggest that the overexpression of the *CYP385C10* contributes to spirodiclofen resistance. Further studies should address in vitro metabolism analyses to better understand resistance mechanisms.

## Figures and Tables

**Table 1:** Probit mortality data for spirodiclofen on the susceptible and resistant *P. citri* strains.

Strain	LC_50_(95%CL) (mg/L)	Slope ± SD	x^2^ (df)	R^2^	RR ^a^
LS	20.09 (16.68–24.19)	1.53 ± 0.48	4.93	0.98	1
DL-SC	14,300 (12,100–16,900)	1.27 ± 0.29	37.32	0.88	712

LS: the laboratory susceptible strain; DL-SC: the field spirodiclofen-resistant strain. ^a^ Resistance ratio (RR) is expressed as the LC_50_ of the resistant strain divided by the LC_50_ of the susceptible strain.

**Table 2:** Detoxifying enzyme activities in the susceptible and resistant *P. citri* strains.

Strain	Specific Activity of P450s ^a^ (nmol/mg Protein/min)	Specific Activity of CCEs ^a^ (nmol/mg Protein/min)	Specific Activity of GSTs ^a^ (μmol/mg Protein/min)
LS	0.96 ± 0.10	31.26 ± 1.02	1.40 ± 0.03
DL-SC	1.47 ± 0.10 *	47.66 ± 1.38 *	1.49 ± 0.38

^a^ Means (± SEM) within a row followed by asterisks indicate significantly different (*t*-test, *p* > 0.05).

**Table 3:** Summary of the transcriptome data for the two *P. citri* strains.

Sample	RawData (bp)	CleanData (bp)	Q20 (%)	Q30 (%)	GC (%)
LS-1	7102549200	7096457818	98.00%	93.65%	36.66%
LS-2	6117744300	6113981009	97.92%	93.50%	37.10%
LS-3	7492022100	7487647999	97.96%	93.65%	36.96%
DL-SC-1	6164956500	6160537642	98.00%	93.66%	36.42%
DL-SC-2	6275241600	6271604780	98.06%	93.81%	36.57%
DL-SC-3	6749435100	6742091542	98.11%	93.99%	36.53%
Average	6650324800	6645386798	98.01%	93.71%	36.71%

**Table 4:** Summary of DEGs annotations.

Gene Name	*P. citri* Gene IDs ^a^	log_2_ratio	*p* Value	ORF (bp)	Description
Cytochrome P450	*EVM0003845*	4.27	1.25 × 10^−35^	1446	cytochrome P450 monooxygenase [*Panonychus citri*]
	*EVM0007673*	4.79	2.04 × 10^−32^	1494	cytochrome P450 monooxygenase [*Panonychus citri*]
	*EVM0001763*	4.90	2.05 × 10^−48^	1500	cytochrome P450 monooxygenase [*Panonychus citri*]
	*EVM0002022*	2.84	3.26 × 10^−9^	1635	CYP391A1 [*Tetranychus cinnabarinus*]
	*EVM0005986*	2.04	1.07 × 10^−16^	1707	CYP391A1 [*Tetranychus cinnabarinus*]
	*EVM0006527*	3.15	5.28 × 10^−5^	1680	probable cytochrome P450 4ac1 [*Tetranychus urticae*]
ABC transporter	*EVM0007979*	1.60	0.0003	1158	ATP-binding cassette sub-family A member 7 [*Tetranychus urticae*]
	*EVM0001067*	1.51	0.001	4518	multidrug resistance-associated protein 1 [*Tetranychus urticae*]
	*EVM0004646*	1.64	0.005	4053	multidrug resistance-associated protein 4-like [*Tetranychus urticae*]
	*EVM0009977*	2.31	1.89 × 10^−5^	4461	multidrug resistance-associated protein 1 [*Tetranychus urticae*]
	*EVM0002879*	1.51	0.0003	2220	ABC transporter G family member 20 [*Tetranychus urticae*]
	*EVM0010399*	1.77	3.85 × 10^−8^	2205	ABC transporter G family member 23 [*Tetranychus urticae*]
	*EVM0000524*	1.98	3.85 × 10^−5^	2280	ABC transporter G family member 20 [*Tetranychus urticae*]
Esterase	*EVM0001285*	1.74	0.0003	1656	acetylcholinesterase-1-like [*Tetranychus urticae*]
	*EVM0004979*	3.17	1.16 × 10^−30^	1635	esterase 5 [*Panonychus citri*]
	*EVM0001466*	1.37	0.0004	1680	esterase 11 [*Panonychus citri*]
Major Facilitator Superfamily	*EVM0010525*	2.77	7.44 × 10^−5^	1359	sodium-dependent glucose transporter 1A-like [*Tetranychus urticae*]
	*EVM0005736*	2.18	0.001	1617	sodium-dependent phosphate transport protein 1-like [*Tetranychus urticae*]
UDP-glucuronosyltransferase	*EVM0011003*	2.00	0.0008	711	demethyllactenocin mycarosyltransferase-like isoform X1 [*Tetranychus urticae*]

^a^*P. citri* gene ID can be accessed at the database (https://ngdc.cncb.ac.cn/search/?dbId=gwh&q=GWHBAOM00000000&page=1 (accessed on 21 October 2021)).

## Data Availability

Transcriptome raw data have been uploaded to NCBI, reference: SRP333472.

## Supplementary Materials

The following supporting information can be downloaded at: https://www.mdpi.com/article/10.3390/insects13111011/s1, Table S1. ACCase gene amplification primers. Table S2. Gene primers for RT-qPCR and RNAi. Figure S1. DiffExp genes statistics. Figure S2. Level 2 GO terms of LS-VS-DL-SC.
