# Supplementary material for: Biochemical and Molecular Analysis of Field Resistance to Spirodiclofen in Panonychus citri (McGregor)
Source: Insects. 2022 Nov 2;13(11):1011. doi: 10.3390/insects13111011 (PMC9696244; doi:10.3390/insects13111011)
Supplement: Supplementary file 1 [file insects-13-01011-s001.zip › Table S1.pdf]

Table S1 *ACCase* gene amplification primers

Genes	Primer Name	Primer sequence (5′ to 3′)	
		F	R
ACCase	ACCase-1	GGGGTGATACTGTTGTT	AGCATTGGCTTTAAGAT
ACCase	ACCase-2	CGGACCTAATCTTTGCTCAC	ATCGGCAAGGAGTTGAACCT
ACCase	ACCase-3	GAAGGAGGTGGAGGTAAAGG	CCACCCGAAGCACTAACACT
ACCase	ACCase-4	AGTGGAACCGTTCAGGAGCT	TTACCTGGTGATGGAGAACG
ACCase	ACCase-5	GGGGAGAAGATCGTGAAG	TCTGTGGGTGATGTGAAT
ACCase	ACCase-6	TCCAACCATTTTACGTTCTCC	GCTTTGCTCATCCCTTCG
ACCase	ACCase-7	TTACGTTCTCCATCACCAGG	CGTAGACGACCACGAATACC
ACCase	ACCase-8	TCGTAACGGTATTCGTGGTC	AAGTGGAAAACTGCTGTTGG
ACCase	ACCase-9	TGGTTGCCTTACAGCATCTT	AAGTGCAGGTTCGAGGTGAC
ACCase	ACCase-10	AACCACTTGCAGCTACCG	CTTTGATTGGCAGTTGGA
ACCase	ACCase-11	GAACCTGCACTTGCCTTCCA	GGCAGTTTGAAACATTACCG
ACCase	ACCase-12	TTTGAGGCTTGGGGTGC	TTTCGGATGTTGGATTTGC
ACCase	ACCase-13	TGTTTCAAACTGCCCTCACT	CTCAACACCAAGGCCCGATT
ACCase	ACCase-14	TAAAGAATCGGGCCTTGGTG	CTTCCGTTTTCCTGGTTTCA
ACCase	ACCase-15	AAGGTGTTTGGGAAGAGGGT	CGATTTGCATTTGCAGCCAT
ACCase	ACCase-16	GCTACCGTTGAGGCTTATTC	TTACTTTGACGAGTCATCCC
