# Supplementary material for: Biochemical and Molecular Analysis of Field Resistance to Spirodiclofen in Panonychus citri (McGregor)
Source: Insects. 2022 Nov 2;13(11):1011. doi: 10.3390/insects13111011 (PMC9696244; doi:10.3390/insects13111011)
Supplement: Supplementary file 1 [file insects-13-01011-s001.zip › Table S2.pdf]

Table S2 Gene primers for RT-qPCR and RNAi

Genes	Primer sequence (5′ to 3′)	
	F	R
ACCase	GAATTTCATCAAATGTCCGAT	TGATTGGCAACAATAACTCG
EVM0007673- qPCR	TTCCCTTCTGGGTTTCATAGC	AACATTGATCGAAGTCGTTTCC
EVM0006527	TCCATCTCGCCGCTAATCT	TCCATCTTCCGTGTAGTCCG
EVM0003845	GAATCAGCCGAAGTTGACAATAC	TGCAATAAGGAAGCTAACCGA
EVM0001763	CGAGAAACGGAAAACTGAGAAA	TGCGATAAGGAAAACGAGTGAA
EVM0006527	TCCATCTCGCCGCTAATCT	TCCATCTTCCGTGTAGTCCG
EVM0002022	CAAATTCATCTTATGTTCGTGCCA	TTCGTTTATAGTATCAATGTCCTTCTG
EVM0005986	CGAAGGAAAATGCTGGAACC	AATATCGAAAACTGAACCAACTGAG
EVM0001285	TGATGATTTCAGTCCCGTTTTC	GATTCTCAGGTAGTCCTTCTTTCG
EVM0004979	CAAATCGACAAGGAAAAGCG	CCAAAATAAAAGCACCACCGT
EVM0001466	GTCCAAAATGTACTCAACTACCGA	GAAACCAAAAGCACCTAAACGA
EVM0007979	TGTTTTGGTCTATTGGGTGTCA	ATCCAGCGATAAAAGCCTCAC
EVM0001067	CGTTCTTGTGGCTTTGCTCT	CCTTTTCTGCTGTCAGTGATTCTT
EVM0004646	TATCGCTTACACGGAAGGTTATG	TTATGTTTCCCGCTTTGTTACTG
EVM0009977	AACTGTTGGTTCTGGTAAATCGTC	ATCCACGCACTTTGAGGCAC
EVM0002879	CAATTATGGAACCTCAGTCAAACC	AGTGTCGTCTTACCGCACCC
EVM0010399	GTGGCAATCACCATCAGCAG	CCATTTGTAGCAGGAGAAGACG
EVM0000524	CATTTGCCTGTTCCCTGGTC	GGTCATTTTCTCGTTCTCCGT
EVM0010525	TATGGGCTGGTTATGGTGGC	TTCCATTGGTCGGTGGTTGT
EVM0005736	TTCAGCAGCTTTGGGATTAGG	TGGTCCAGCGACAGTAAGAGTAG
EVM0011033	CGCAAGATTTTGAGTTACCCG	TCGACCAGCATTTTAATGAGC
ELF1A	GGCACTTCGTCTTCCACTTC	ATGATTCGTGGTGCATCTCA

GADPH	CTTTGGCCAAGGTCATCAAT	CGGTAGCGGCAGGTATAATG
EGFP-RNAi	TAATACGACTCACTATAGGGCCACAAGTTCAGCGTGTCC	TAATACGACTCACTATAGGGTGGGTGCTCAGGTAGTGGTTGT
EVM0007673- RNAi	TAATACGACTCACTATAGGGTGTCAAAGGTCCAAAGC	TAATACGACTCACTATAGGGATTAGCGGCGAAACAAC
