# Supplementary figures and images for: Biochemical and Molecular Analysis of Field Resistance to Spirodiclofen in Panonychus citri (McGregor)
Source: Insects. 2022 Nov 2;13(11):1011. doi: 10.3390/insects13111011 (PMC9696244; doi:10.3390/insects13111011)

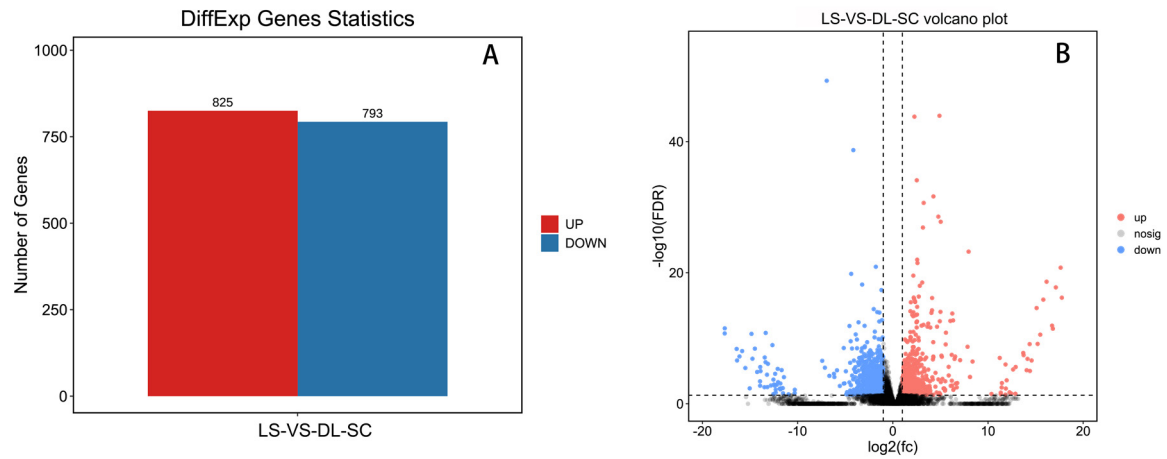

**Figure S1.** DiffExp genes statistics.

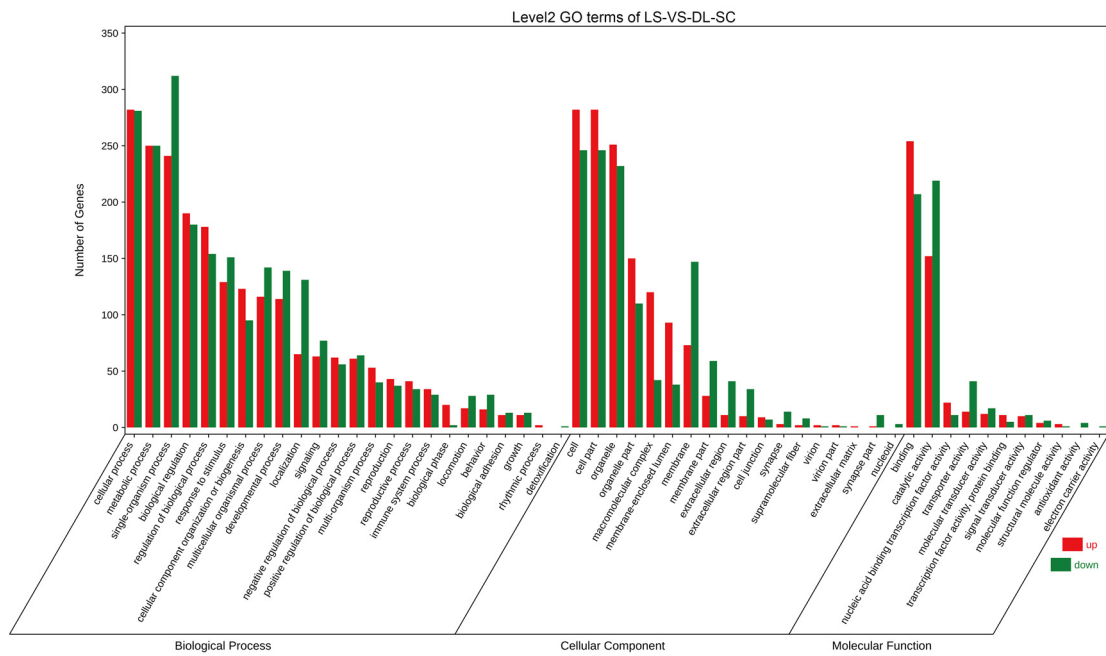

**Figure S2.** Level 2 GO terms of LS-VS-DL-SC.

Supplement: Supplementary file 1 [file insects-13-01011-s001.zip › Figures S1 and S2.pdf]
